# Flavor science in the context of research on electronic cigarettes

**DOI:** 10.3389/fnins.2022.918082

**Published:** 2022-07-27

**Authors:** John E. Hayes, Allison N. Baker

**Affiliations:** ^1^Sensory Evaluation Center, College of Agricultural Sciences, The Pennsylvania State University, State College, PA, United States; ^2^Department of Food Science, College of Agricultural Sciences, The Pennsylvania State University, State College, PA, United States; ^3^Interdepartmental Neuroscience Program, Huck Institutes of Life Sciences, The Pennsylvania State University, State College, PA, United States

**Keywords:** smoking, flavor, smell, taste, chemesthesis, perception, sensory evaluation

## Abstract

Thousands start smoking or vaping daily, despite long-standing efforts by public health authorities to curb initiation and use of nicotine containing products. Over the last 15 years, use of electronic nicotine delivery systems has increased dramatically, with a diverse range of products on the market, including pod-based, disposable, and refillable electronic cigarettes (eCigs). Originally intended for harm reduction and smoking cessation, eCigs may encourage nicotine use among never smokers, given the vast range of appealing flavors that are available. To better understand abuse liability and to facilitate appropriate regulations, it is crucial to understand the science of flavor, and flavor perception within the context of eCig use. Here, we (a) provide a brief primer on chemosensory perception and flavor science for addiction and nicotine researchers, and (b) highlight existing some literature regarding flavor and nicotine use, with specific attention given to individual differences in perception, and interaction between different sensory modalities that contribute to flavor. Dramatic increases in use of eCigs highlights the importance of flavor science in contemporary addiction research, both with regards to public health and regulatory efforts. Other recent work summarizes findings on flavored e-liquids and eCig use, but none have focused explicitly on chemosensory processes or flavor perception as they relate to appeal and use of such products. We argue flavor science needs to be considered as perceptual and behavioral phenomena, and not merely from analytical, toxicological and pharmacological perspectives; we help address this gap here.

## Introduction

Nicotine use is a major public health concern, with smoking being the leading preventable cause of death in the United States (CDC, 2014). Despite decades of public health efforts intended to prevent initiation, thousands of young people take up smoking each day. Since the introduction in electronic cigarettes (eCigs) in the United States in 2007 and the United Kingdom in 2012, their use has increased dramatically, and thousands of flavors are currently available in a variety of categories. While combustible tobacco cigarettes and pod-based eCigs are highly regulated in the United States and cannot contain characterizing flavors (beyond menthol), disposable eCigs are not currently covered under these regulations (Bernat et al., 2021; Ramamurthi et al., 2022). Further, flavored e-liquids for refillable devices are remain widely available in the United States, although some states and localities have enacted their own bans (Posner et al., 2022). The extensive and highly appealing range of flavors—including fruit, dessert, cocktail themed flavors—can be a major factor in many individuals’ decision to try and use eCigs.

The public health implications of eCigs are complex and nuanced, given competing issues of harm reduction for smokers vs. potential risk of initiation in non-users. On one hand, eCigs may play an important role in harm reduction by helping current smokers eliminate or reduce use of combustible tobacco. For example, ambivalent smokers randomized to tobacco or menthol flavored eCigs that deliver nicotine doses comparable to combustible cigarettes were more likely to quit smoking entirely at 24 weeks, relative to ambivalent smokers randomized to placebo or lower dose eCigs, with the caveat that overall quit rates were quite low (Foulds et al., 2022). Elsewhere, data from young adult binge drinkers suggest that when comparing baseline to a 24-month follow-up, most exclusive eCig users transitioned to abstinence or remained as exclusive eCig users; notably, none transitioned to exclusive combustible tobacco use or dual use. Further, among those who were dual users at baseline, most transitioned to non-use (41%), or exclusive eCig use (24%), or remained dual users (17%), leading the authors to conclude that concurrent or exclusive eCig use was not a risk factor for development or persistence of combustible tobacco use (Martinez-Loredo et al., 2022). Conversely, among never smokers, eCig use may represent a novel on-ramp leading to development of nicotine dependence, especially for adolescents and young adults who find the diverse flavors of eCigs highly appealing. For example, in a meta-analysis from 2017, prior eCig use substantially increased the risk of smoking initiation among adolescent or young adult never smokers (pooled OR of 3.5), with a caveat that the longitudinal studies analyzed largely predated the introduction of pod-based eCigs. When creating public health policies, it is important to balance needs of current cigarette smokers (i.e., harm reduction), with a strong need to minimize initiation of new nicotine use in non-smokers (Selya and Foxon, 2021).

By 2013 (i.e., within a few years of market introduction), ever use of eCigs among adults in the United States was estimated to be 8.5% (King et al., 2015), with higher usage among young adults, women, and current smokers (see Berg, 2016). Regarding new initiation in non-smokers, data from a 2018 Monitoring the Future survey were especially worrisome: among high school seniors, the proportion who reported vaping in the last 30 days almost doubled, from 11 in 2017 to 20.9% in 2018 (MTF, 2018). Fortunately, subsequent data from the National Youth Tobacco Survey comparing 2019 and 2020 showed substantial drops in last 30 day use for both middle school (10.5% to 4.7%) and high school (27.5% to 19.6%) students in the United States (Choi and Abraham, 2021). Still, NYTS data suggest over three quarters of these adolescents prefer sweet fruit flavored eCigs. Thus, with added flavors featuring prominently in the use experience from eCigs and other products (e.g., Owens et al., 2019), addiction researchers may benefit from a more nuanced understanding of chemosensation and flavor perception.

Many reports explore relationships between smoking and chemosensation, but much of this work focuses on the effects of smoking on taste or smell rather than asking how flavor might affect use of nicotine containing products. An early 1961 example investigated impairment of taste in smokers, finding bitterness was altered in smokers relative to non-smokers, while taste qualities like sweet, sour, and salty did not differ (Krut et al., 1961), a result that was confirmed a half century later (Jacob et al., 2014). Elsewhere, nationally representative data from the National Health and Nutrition Examination Survey (NHANES) show chronic smoking associates with smell dysfunction (Glennon et al., 2017) and taste dysfunction (Berube et al., 2021). These are just a few examples of many cross sectional and longitudinal studies showing adverse effects of combustible tobacco use on chemosensory function. Critically, however, influences of flavor on use of nicotine containing products is a separate question, which is the focus of the present work.

Specifically, we argue *flavor* is a critical factor in the appeal of eCigs, as noted elsewhere (e.g., Baker et al., 2021b; Bernat et al., 2021; Pang et al., 2022; Ramamurthi et al., 2022). For example, one early study found, on average, individuals used three different types of e-liquid on a regular basis, and exclusive vapers switched flavors more frequently than those who concurrently vape and smoke (Farsalinos et al., 2013). In their profile of vapers, Dawkins et al. (2013) found individuals start using flavored eCigs because they wanted either a complete or partial alternative to smoking, due to curiosity, or because of a friend’s recommendation.

Characterizing flavors in cigarettes (other than menthol) have been prohibited in the United States since 2009 and the European Union since 2014. However, eCigs have exploded in popularity since their introduction, and there are thousands of flavors currently on the market in the United States and Europe. By 2014, 466 e-liquid brands and over 7,000 unique flavors were available (Zhu et al., 2014). In 2016, Berg reported fruit flavors were preferred among current eCig users, but flavor preferences varied among never, current, and former smokers (Berg, 2016). Soule et al. (2016) used concept mapping to explore reasons for using flavored e-liquids; reasons identified by participants included increased satisfaction/enjoyment, better feel/taste than cigarettes, variety/customization, food craving suppression, and social impacts. Laboratory data in young smokers suggest flavored eCigs have greater subjective reward and reinforcing value, relative to unflavored eCigs, which may increase abuse liability (Audrain-McGovern et al., 2016). While nicotine is clearly the primary reinforcer in tobacco and eCigs, added flavors may also act as rewards and/or reinforcers in their own right (Patten and De Biasi, 2020; Cooper et al., 2021). Animal data support the idea that characterizing flavors (and menthol) can modulate circuits involved in reward, reinforcement and motivation (Cooper and Henderson, 2020), possibly by modulating nicotinic acetylcholine receptors directly (Henderson et al., 2018).

Accordingly, some countries have outright (e.g., India) or functional (e.g., Japan) bans on eCigs, while others have moved to ban eCig flavors other than tobacco (e.g., Denmark, the Netherlands). Still, some policy experts have suggested bans on all non-tobacco flavors may not be appropriate, as flavored eCigs may still play a role in harm reduction for current smokers (Bauld and Gage, 2019). For example, Dawkins et al. (2013) reported that over 90% of their respondents reported substantial reductions in tobacco craving with eCig use. When non-treatment seeking cigarette smokers in a 6 week invention were randomized to eCigs in four flavors, tobacco, menthol, cherry or chocolate, combustible tobacco use and breath carbon monoxide levels dropped for all participants, and vaping rates were greatest for the most preferred flavors (Litt et al., 2016).

In 2016, the United States FDA extended its tobacco regulatory authority to include electronic nicotine delivery systems, including eCigs (Backinger et al., 2016). In early 2020, updated guidance from FDA functionally banned the sale of pod-based or cartridge-based eCigs in flavors other than tobacco or menthol (Bernat et al., 2021). Critically, however, disposable eCigs (like PuffBar) were not covered under this updated guidance (Ramamurthi et al., 2022), so flavored eCigs remain widely available in the United States. Further, flavored e-liquids for use in refillable tank-style vape pens and box mods also remain widely available in the United States.

Given a need to enhance the evidence base in support of updated regulations, we felt it might be useful to elaborate on the potential role of flavor in the initiation and/or use of eCigs, toward better policy, by leveraging our expertise in flavor science. Specifically, we (a) provide a brief primer on chemosensation and flavor perception for addiction and nicotine researchers, (b) review individual differences in sensation that may be potentially relevant, and (c) discuss perceptual interactions that occur when participants are given chemically complex stimuli that activate more than one sensory modality. Select examples of research on flavor perception and use of combustible cigarettes and eCigs are woven throughout; these highlighted examples are not intended to be exhaustive.

## Fundamentals of chemosensation

**FIGURE 1:**
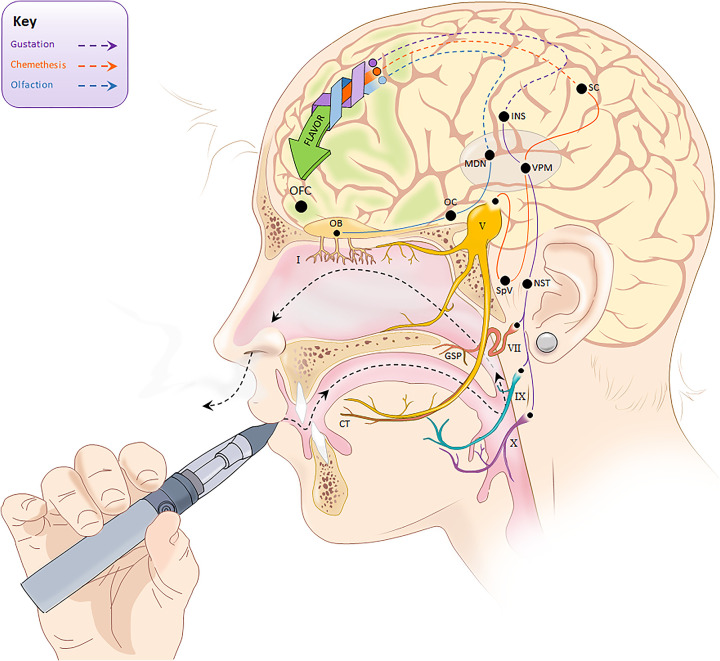
Summary of three distinct sensory pathways involved in flavor perception. Taste, smell, and chemesthesis are integrated in the orbitofrontal cortex (OFC) to generate the unitary percept that we call flavor. Taste signals are carried by Cranial Nerves VII, IX, and X to the nucleus of the solitary tract (NST), which connects to the Ventral Posteromedial Nucleus (VPM) within the Thalamus. The VPM projects to the taste cortex located in the Insula. CN VII (the facial nerve) has two branches involved in taste: the chorda tympani (CT) innervates the anterior tongue while the greater superficial petrosal nerve (GSP) innervates the palate. Smell signals are carried by Cranial Nerve I through the cribriform plate to the olfactory bulb (OB), the Olfactory Cortex (OC), and then the Medial Dorsal Nucleus (MDN) within the Thalamus. Chemesthetic signals are carried by multiple cranial nerves (see Green, 2016), but for simplicity, only the trigeminal nerve (CN V) is shown here. Separate branches of the trigeminal nerve come together in the trigeminal ganglion (not shown), before descending to the spinal trigeminal nucleus (SpV) in the brain stem. From the SpV, signals ascend contralaterally to the VPM in the Thalamus, and then to the Somatosensory Cortex (SC). (Some anatomical landmarks in the brain have been omitted, and positions shown here may not be exact).

By the early twentieth century, numerous researchers had recognized combined inputs from the taste, smell, and touch systems give rise to integrated percepts when we eat or drink. In 1982, Rozin remarked that the word “*flavor”* best captures the combination of oral and olfactory sensations we perceive with ingestion of most foods, at least in English (Rozin, 1982). Today, most Neuroscientists, Sensory Psychologists, and Sensory and Consumer Scientists define *flavor* as the unitary percept which coalesces from the integration of *smell*, *taste*, and *chemesthesis* in the orbitofrontal cortex (Lawless, 1996; Small and Prescott, 2005; Prescott, 2012; Heymann, 2019). Despite this broad consensus, there remains some degree of confusion around these terms, regarding their colloquial and technical usage, even within medical professionals (Boltong et al., 2011a,b), so each of the three sensory modalities that contribute to flavor will be briefly detailed here. The pathways for each are summarized in Figure 1.

Olfaction (smell) occurs when we sense volatile chemical messages from the environment (via the nares) or from the oral cavity (through the back of the throat). Odor active volatiles (i.e., odorants) activate specialized G-protein Coupled Receptors (Persuy et al., 2015) expressed in olfactory sensory neurons (OSNs) found near the top of the nasal cavity. When an odorant binds to specialized receptor proteins expressed on the surface of OSNs, it initiates a transduction cascade which converts the chemical signal into an electrical signal. The ensuing action potential is carried by the axon of the olfactory neuron through the cribriform plate, where the axons synapse onto second-order neurons in the olfactory bulb (see review by Duffy and Hayes, 2014). Because cell bodies of the OSNs sit at the top of the nasal cavity, below the cribriform plate, they are easily damaged by pollutants, viruses and toxins (including tobacco smoke). However, OSNs are continually replaced, roughly every 30 days, which preserves function despite such environmental insults. In contrast to other senses, smell is a dual sensory modality: that is, it occurs either orthonasally or retronasally and this affects where we localize the percept. Ecologically speaking (Gibson, 1966), orthonasal olfaction is an external sense focused on objects and information in the environment, while retronasal olfaction is an internally focused sense where volatiles that reach the olfactory epithelium via the pharyx during chewing or swallowing are perceived as being present in the mouth.

Gustation (taste) occurs when non-volatile chemical stimuli dissolve in saliva and contact specialized taste receptor cells (TRCs) found in the tongue, soft palate and throat. Unlike the OSNs mentioned above, the TRCs are not neurons—rather, they are specialized epithelial cells which must communicate with neurons to project a signal centrally. (A discussion of the different types of TRCs is beyond the scope of this review). Taste aids organisms in perception of nutrients and toxins, driving ingestion via affective responses (Breslin and Spector, 2008; Boesveldt and de Graaf, 2017). The widely accepted prototypical taste qualities are sweet, salty, sour, bitter, and savory/umami (the meaty taste of certain amino acids). Non-sweet starch taste, fatty acid taste (oleogustus), metallic taste, and astringent may also be distinct taste qualities, but the case for each is less clear and their inclusion as distinct qualities is still actively debated. Individuals vary widely in terms of taste perception, due in part to genetic variation (e.g., Wooding et al., 2010; Hayes et al., 2015). Such differences are potentially important for nicotine research, and are discussed more below.

Chemesthesis is the sensibility that results from chemical stimulation of somatosensory nerves (Green, 1996; Lawless, 1996); that is, it can be thought of as chemically initiated touch (McDonald et al., 2016). Chemesthetic stimuli have a range of perceptual qualities, including the tingling elicited by carbonation, the burn from chili peppers, the burn from horseradish, the mechanical buzzing from Sichuan Buttons, and best known to tobacco researchers, the cooling from menthol. As chemesthetic stimuli are known to trigger cough reflexes (Wise et al., 2012), they have strong relevance to eCigs, especially given the importance of irritation or throat hit to eCig liking and appeal (e.g., Goldenson et al., 2016; Mead et al., 2019; Baker et al., 2021a). Extensive discussions of menthol as it relates to use of nicotine containing products are covered in detail elsewhere (e.g., Rose, 2006; Mangold et al., 2008; FDA, 2013; Oncken et al., 2015; DeVito and Krishnan-Sarin, 2018), so comments below will be restricted to specific aspects related to narrowly to chemosensation.

Notably, the classical assumption that nicotine is itself bitter is almost certainly in error. Rather, three distinct and complementary lines of evidence suggest nicotine gives rise to chemesthetic sensations, rather than bitterness *per se*. First, in heterologous expression systems, nicotine does not activate any known bitter taste receptor (Meyerhof et al., 2010), but it does activate TRPA1 (Talavera et al., 2009), a receptor activated by ligands like cinnamaldehyde or allyl isothiocyanate (AITC) that impart the pungency of cinnamon and wasabi, respectively. Second, electrophysiology data from rats (Dessirier et al., 2000) and psychophysical data from humans (Carstens et al., 2007) each indicate nicotine is a chemesthetic stimulus. Third, close reading of very old literature suggests a widely cited 1959 source for the widespread claim that nicotine is bitter in turn leads back to an earlier paper from 1885. Critically, if one reads the original source from 1885, the authors explicitly write “nicotine does not trigger a taste sensation,” noting that if the concentration is increased, it produces “*a stinging sensation, which is not, strictly speaking, a taste sensation, but tactile*” (see discussion in Gyekis et al., 2012). This caveat notwithstanding, combustible tobacco smoke certainly gives rise to bitter sensations from one of the hundreds of other compounds found in smoke, but strictly speaking it does not seem such bitterness can be directly attributable to nicotine. Regarding eCigs, participants report bitterness in multiple studies, but the source of this bitterness remains unknown.

## Individual differences in taste perception due to normal genetic variation

Bitterness is innately aversive, so bitterness from nicotine products is presumably a deterrent to initiation of use. Ligands humans describe as bitter are sensed by specialized G-Protein Coupled Receptors (GPCRs) encoded by *TAS2R* genes. In humans, there are ∼25 *TAS2Rs* that encode functional receptors, and 5 of these genes show non-synonymous polymorphisms which alter receptor function, resulting in differential taste phenotypes (e.g., Meyerhof et al., 2010; Risso et al., 2014; Hayes et al., 2015) which are sufficient to influence ingestive behavior. Regarding eCigs, multiple recent studies suggest greater bitterness predicts lower liking (Kim et al., 2016; Pang et al., 2022).

If bitterness acts as a barrier against initiation and/or persistence, then it follows individuals who experience less bitterness due to genetic polymorphisms in *TAS2Rs* may have a lower barrier to early use. The suggestion that smoking behavior may vary with taste phenotype is not new, as it dates back to the 1960s (Kaplan et al., 1964; Kang et al., 1967), although findings are highly mixed. For example, Enoch, Harris and Goldman reported a lower proportion of individuals sensitive to phenylthiocarbamide (a bitterant commonly used for taste phenotyping) among smokers compared to non-smokers and social smokers (Enoch et al., 2001). Similarly, Snedecor et al. (2006) tested whether smokers who taste phenylthiocarbamide as bitter differed from smokers who find it to be tasteless. Compared to population norms, there were substantially fewer phenylthiocarbamide tasters in their smokers (33 vs. 70%), and years smoked, nicotine dependence (from the Fagerstrom Tolerance Questionnaire) and the positive reinforcement value of smoking (from the Michigan Nicotine Reinforcement Questionnaire) each differed between bitter sensitive and bitter insensitive individuals.

Cannon et al. (2005) reported data that would initially appear to be consistent with the protection hypothesis, as they found polymorphisms in the *TAS2R38* bitter receptor gene associated with smoking. However, a close reading reveals haplotypes associated with lower smoking incidence were not the ones that would be predicted *a priori*, so these data fail to support the protection hypothesis. Elsewhere, Risso et al. (2016) found expected associations between common *TAS2R38* haplotype and smoking status in European Americans, but not in African Americans, while Mangold and coworkers found the non-taster haplotype as associated with nicotine dependence in African American women but not in European Americans (Mangold et al., 2008). In a crowdsourced sample, Baker et al. (2018) found a significant relationship between propylthiouracil (PROP) bitterness and smoking status, but the effect was the opposite of what was expected: current smokers perceived higher, not lower, bitterness than never smokers. Moreover, there was no relationship between *TAS2R38* haplotype and smoking status. In summary, whether looking at thiourea taste phenotype or *TAS2R38* genotype, extant data are highly conflicted, with no obvious explanation for the discordant results across studies and samples and studies.

Notably, notwithstanding a single study that included *TAS2R16* variants, functional polymorphisms in other bitter genes beyond *TAS2R38* (i.e., *TAS2R4*, *TAS2R13*, and *TAS2R31*) have not been studied in relation to use of combustible tobacco or eCigs. Even if a direct influence of *TAS2R* variants on initiation and/or use fails to emerge as a robust finding in the future, there may still be indirect effects vis-à-vis comorbidity with alcohol use, misuse, and abuse (e.g., Grant et al., 2004). That is, because *TAS2R38* alleles robustly associate with differential bitterness and liking of ethanol and alcohol intake (Hayes et al., 2011; Dotson et al., 2012; Allen et al., 2014; Nolden et al., 2016; Beckett et al., 2017), reports suggesting *TAS2R* variants may associate with both tobacco and alcohol use (e.g., Keller et al., 2013) are not surprising, given the frequency with which alcohol and nicotine are used together. Still, while bitterness may differentially deter bitter sensitive individuals from using combustible cigarettes, other means of delivery may or may not evoke bitterness to the same degree, so potential influences of bitter taste phenotype or *TAS2R* genotype on initiation and use of eCigs requires additional research. One recent study found within-participant differences in bitterness elicited by flavored eCigs predicted their appeal (Pang et al., 2022); unfortunately, the authors did not genotype their participants for functional *TAS2R* alleles, so whether this variation might have a genetic basis remains an open question. Other recent work suggests bitter taste phenotype may influence liking of eCig flavors differentially—while bitterness from eCigs did not vary by PROP phenotype, liking ratings did, and those who experienced the most bitterness from PROP reported the highest liking ratings for menthol eCigs (Mead et al., 2019). Accordingly, more work in this area appears warranted.

## Key interactions within and between chemosensory modalities

When considering the flavor of eCigs and other nicotine products, it may be helpful to understand two well-known phenomenon—*mixture suppression*, and *cross-modal modulation*—that alter the perception of complex stimuli relative to percepts from simple model systems containing a single stimulus. Both of these phenomena have potentially important implications for sensory and affective responses to flavored eCigs.

When a bitter tastant (like quinine) is mixed with a sweet tastant (like sucrose) in an equimolar mixture, the sweetness and bitterness of the mixture is less than either one would be in isolation: this is known as *mixture suppression* (Lawless, 1977, 1979; Kroeze and Bartoshuk, 1985). Critically, this effect can be observed in real world stimuli, not just model systems, when careful psychophysics are used (e.g., Bakke et al., 2018; Higgins and Hayes, 2020). A similar pattern of hypo-additivity is also seen when two qualitatively distinct odorants are mixed—a blend of lavender (floral) and pyridine (fish-like) results in lower ratings of each relative to either one presented in isolation. In a controlled psychophysical experiment with adult smokers given a V2 eCig (Rosbrook and Green, 2016), nicotine enhanced, rather than suppressed, cool sensations, suggesting a possible synergistic effect between nicotine and menthol. For harshness, a complex interaction was observed: there was an analgesic effect at high menthol *and* high nicotine concentration, but menthol by itself also contributed to irritation. This suggests menthol provides some protection against irritation, and reduces disliked sensations from inhaled nicotine (Rosbrook and Green, 2016), consistent with the idea that menthol increase risk of addiction by increasing tolerance of unpleasant sensations of smoking (e.g., Oncken et al., 2015).

As shown in Figure 1, flavor perception occurs following integration of signals from three physiologically distinct sensory modalities into a single unitary percept in the orbitofrontal cortex (Small and Prescott, 2005; Shepherd, 2006). Because the resulting percept arises from multiple modalities, it should not be surprising that *cross-modal modulation* (or interaction) is an extremely common and well documented phenomenon. For example, vanilla extract or vanillin increase the perceived sweetness of model systems (Labbe et al., 2006b) and real beverages in adults (Labbe et al., 2006a) and in children (Lavin and Lawless, 1998) using a variety of methods (Wang et al., 2018, 2019). Likewise, fruity smelling odorants like ethyl butyrate show enhancement of perceived sweetness (Labbe et al., 2006b). The olfactory contribution to this enhancement is illustrated by work showing the increase in sweetness from maltol disappears when the nostrils are pinched closed during tasting (Bingham et al., 1990). A related compound, ethyl maltol, smells like cotton candy/candy floss or cooked, caramelized sugar, so the finding that ethyl maltol is a common constituent in e-liquids (Miao et al., 2016) is not a surprise. Other evidence indicates *mixture suppression* and *cross-modal modulation* may potentially interact with each other to further modify flavor perception. For example, Isogai and Wise found sweet smelling odorants like ethyl hexanoate and vanillin were able to significantly reduce the intensity of a bitter tastant and a bitter smelling odorant was able to suppress the sweetness of a sucrose solution (Isogai and Wise, 2016); that is, *mixture suppression* can occur cross-modally between taste and smell.

In a study of experienced eCig users given a non-refillable cigalike eCig in six different flavors, sweetness and bitterness varied significantly by flavor, and notably, the sweetest flavor (Piña Colada) was the least bitter, and the second sweetest flavor (Peach Schnapps) was the 2nd least bitter (Kim et al., 2016). As commercial e-liquids were used, it is not possible to rule out that the formulas may have differed in other ways, but the patterns seen are wholly consistent with what one would expect from cross-modal enhancement of sweetness by smell, and subsequent mixture suppression of bitterness. Notably, harshness did not vary by flavor. In a separate study where e-liquids were custom formulated for use in a tank-based or cartridge-based eCig, when sucralose, a high potency non-nutritive sweet tastant, was included in the e-liquid, chemical analysis revealed a higher concentration of sucralose in the eCig vapor from the cartridge-based eCig. When subsequently given in a controlled psychophysical experiment with and without added sucralose, adding sucralose enhanced perceived sweetness across all 4 flavor conditions (Rosbrook et al., 2017). However, harshness ratings were not significantly depressed by addition of sucralose, although this might reflect a floor effect, as harshness ratings from the cartridge based eCigs were quite low (mean below “weak”). When given 10 different flavors that included flavors categorized as sweet or non-sweet by the researchers, non-treatment seeking vapers rated the sweet flavors as sweeter than non-sweet flavors, which were still sweeter than the no flavor condition (Goldenson et al., 2016), but flavor condition did not influence ratings of throat hit. Finally, when Pullicin et al. (2020) gave current eCig users V2 eCigs with varying amounts of nicotine, a greater concentration increased irritation ratings (as expected) and more notably, significantly depressed sweetness ratings. Collectively, it seems sweet smelling e-liquids increase sweetness and decrease bitterness, but potential influences on harshness/throat hit are less clear. For additional discussion on addition of sweetness, see Goldenson et al. (2016), Patten and De Biasi (2020).

Unsurprisingly, these kinds of perceptual interactions also have downstream influences on hedonic (affective) responses. In the case of mixture suppression, hedonic shifts are seen even when an aversive sensation is still perceptible in the mixture (Lawless, 1977). For example, a 10 μM quinine solution is unpleasant by itself, but this unpleasantness decreases as sucrose is added, and samples with sufficient added sucrose are positively valanced, despite still being perceptibly bitter. Likewise, lavender/pyridine mixtures smelled orthonasally will still generate positive pleasantness ratings, if sufficient lavender is added. Fifty years ago, Cain and Drexler distinguished between mixture suppression (which they called odor counter-action) and masking (Cain and Drexler, 1974), with the former being reduction of the intensity of a malodor to make it acceptable, and the latter being modification of the perceived quality of an odor to make it more acceptable.

Subsequently, Lawless showed not only that the components of a mixture are less intense when combined, but also that mixing them directly influences pleasantness (Lawless, 1977). That is, if a pleasant stimulus is added to an unpleasant stimulus, there is a shift in pleasantness, and some of this is due to the reduction of the intensity of the unpleasant quality (i.e., an indirect effect due to mixture suppression), while some of the change in pleasantness is added directly, by the simple presence of the pleasant stimulus itself. This framework may be highly relevant when studying affective responses to nicotine containing products with added flavors. Specifically, in a small lab based vaping study, cherry flavor appeared to increase liking of the high nicotine condition directly, without increasing ratings of sweetness or depressing ratings of harshness, leading the authors to conclude the increased acceptability of the cherry condition (despite the high nicotine level) was due to direct addition of pleasantness by the cherry flavor (Baker et al., 2021a). Elsewhere, adding vanillin to ethanol has been shown to increases liking without altering burn (Gaby et al., 2019). Conversely, when nicotine-free propylene glycol/vegetable glycerine (PG/VG) mixtures were co-presented with fruity or confectionary-associated odorants (iso-amyl acetate, ethyl butyrate, vanillin, and ethyl maltol) in young adults who did not regularly use nicotine containing products, the fruity aromas (iso-amyl acetate, ethyl butyrate) increased sweetness but not pleasantness, while the confectionary aromas (vanillin, ethyl maltol) increased pleasantness but not sweetness. Other reports also find that sweetness and throat hit/irritation are positive and negative predicts of appeal in vapers (Goldenson et al., 2016; Kim et al., 2016), while acknowledging that throat hit/irritation may be a positive attribute in dual users who also smoke combustible tobacco (Pullicin et al., 2020).

Collectively, it seems likely adding highly liked flavors may increase liking for otherwise unpleasant sensations like bitterness, harshness, and throat hit, via mixture suppression. That is, unflavored eCig vapor may be bitter or harsh but these unpleasant sensations are reduced (masked) if another flavor is added, with downstream impact on affective responses. Alternatively, however, an added flavor may not directly modulate aversive sensations from the eCig, but instead make the overall experience less negative via pleasantness arising directly from addition of a highly liked flavor. Thus, more work teasing apart which mechanism predominates seems worthwhile, as it may influence policy choices. For example, if direct addition of pleasantness predominates, then restricting eCig flavors to traditional tobacco may substantially address concerns about initiation of use in non-smokers.

## Summary and conclusion

Combustible cigarettes and eCigs are not merely rapid and convenient means to deliver a pharmacological agent like nicotine—rather, flavor appears to be an important but understudied component of nicotine use, as it may be reforcing or rewarding on its own. Given the rapid growth in the popularity of eCigs over the last 15 years, especially among developmentally vulnerable adolescents, it is necessary to gain a better understanding of the role flavor has in both initiation of use in non-users and persistence of use among long-term users, as well as the potential for improved cessation efficacy among those who already smoke. Individual differences in perception due to normal genetic variation may differentially protect some individuals from use but existing data conflict, with no obvious explanation for conflicting results. Further, it remains unknown whether added flavors reduce aversive sensations from eCigs via mixture suppression, or if other flavors improve the appeal of eCigs beyond tobacco or menthol flavors because these other flavors are appealing in and of themselves: both mechanisms may be at play. Flavor science is a mature field, and the tobacco industry has actively employed sensory scientists for over half a century (see Patten and De Biasi, 2020). Addiction researchers working on combustible tobacco, eCigs, and other electronic nicotine delivery systems may benefit from incorporating psychophysical and chemosensory expertise into their work.

## Author contributions

JH: conceptualization, supervision, drafting, revision, and submission. AB: conceptualization and drafting. Both authors contributed to the article and approved the submitted version.

## Conflict of interest

AB is employed by Curion, a research service provider to the Food, Beverage, and FMGC industries, where she manages taste tests on chocolate and confectionary products; Curion has no other interest or involvement in this work. JH has received speaking and/or consulting fees from non-profit organizations, corporate clients in the food and beverage industries, and federal agencies, including the United States Food and Drug Administration Center for Tobacco Products. The FDA CTP was not involved in this project and the views shown here belong solely to the authors. JH also serves as Director of the Penn State Sensory Evaluation Center, which conducts routine taste tests for food industry clients to facilitate experiential learning for undergraduate and graduate students. JH holds equity in Redolynt LLC, which he cofounded in 2021; Redolynt has no involvement in the work described here. None of the organizations listed above have had any role in the conception, drafting or decision to publish this work.

## Publisher’s note

All claims expressed in this article are solely those of the authors and do not necessarily represent those of their affiliated organizations, or those of the publisher, the editors and the reviewers. Any product that may be evaluated in this article, or claim that may be made by its manufacturer, is not guaranteed or endorsed by the publisher.
